# Repurposing SGLT2 Inhibitors for Neurological Disorders: A Focus on the Autism Spectrum Disorder

**DOI:** 10.3390/molecules27217174

**Published:** 2022-10-23

**Authors:** Mohammed Moutaz Nakhal, Salahdein Aburuz, Bassem Sadek, Amal Akour

**Affiliations:** 1 Department of Biochemistry, College of Medicine and Health Sciences, Al-Ain P.O. Box 15551, United Arab Emirates; 2 Department of Pharmacology and Therapeutics, College of Medicine and Health Sciences, Al-Ain P.O. Box 15551, United Arab Emirates; 3 Zayed Center for Health Sciences, United Arab Emirates University, Al-Ain P.O. Box 17666, United Arab Emirates; 4 Department of Biopharmaceutics and Clinical Pharmacy, School of Pharmacy, The University of Jordan, Amman 11942, Jordan

**Keywords:** autism spectrum disorder, sodium-glucose cotransporter 2 inhibitors, canagliflozin, neurological disorders, oxidative stress, anti-inflammatory

## Abstract

Autism spectrum disorder (ASD) is a neurodevelopmental disorder with a substantially increasing incidence rate. It is characterized by repetitive behavior, learning difficulties, deficits in social communication, and interactions. Numerous medications, dietary supplements, and behavioral treatments have been recommended for the management of this condition, however, there is no cure yet. Recent studies have examined the therapeutic potential of the sodium-glucose cotransporter 2 (SGLT2) inhibitors in neurodevelopmental diseases, based on their proved anti-inflammatory effects, such as downregulating the expression of several proteins, including the transforming growth factor beta (TGF-β), interleukin-6 (IL-6), C-reactive protein (CRP), nuclear factor κB (NF-κB), tumor necrosis factor alpha (TNF-α), and the monocyte chemoattractant protein (MCP-1). Furthermore, numerous previous studies revealed the potential of the SGLT2 inhibitors to provide antioxidant effects, due to their ability to reduce the generation of free radicals and upregulating the antioxidant systems, such as glutathione (GSH) and superoxide dismutase (SOD), while crossing the blood brain barrier (BBB). These properties have led to significant improvements in the neurologic outcomes of multiple experimental disease models, including cerebral oxidative stress in diabetes mellitus and ischemic stroke, Alzheimer’s disease (AD), Parkinson’s disease (PD), and epilepsy. Such diseases have mutual biomarkers with ASD, which potentially could be a link to fill the gap of the literature studying the potential of repurposing the SGLT2 inhibitors’ use in ameliorating the symptoms of ASD. This review will look at the impact of the SGLT2 inhibitors on neurodevelopmental disorders on the various models, including humans, rats, and mice, with a focus on the SGLT2 inhibitor canagliflozin. Furthermore, this review will discuss how SGLT2 inhibitors regulate the ASD biomarkers, based on the clinical evidence supporting their functions as antioxidant and anti-inflammatory agents capable of crossing the blood-brain barrier (BBB).

## 1. Introduction

Autism spectrum disorder (ASD) is a common neurodevelopmental disorder that affects about 1% of the population [1]. The reported global prevalence of ASD is one in 161 people, while in the UAE it affects one in 89 people [2,3]. The reported incidence of ASD has increased substantially in recent years, as it is estimated that one child is diagnosed with ASD every 20 min in the United Arab Emirates [2,3]. ASD is characterized by repetitive behavior, deficits in interactions, social communication, and activities [1]. Currently, there is no definitive cure for this disorder, and most therapies aim at ameliorating the symptoms, improving the children’s functioning and supporting their learning and development. In fact, various pharmacological agents, dietary therapies, and behavioral interventions have been utilized to benefit this condition [4]. Sodium-glucose cotransporter 2 (SGLT2) inhibitors are considered as oral glucose-lowering medication that work by inhibiting the reabsorption of the renal glucose [5]. The list of SGLT2 inhibitors that are Food and Drug Administration (FDA) approved, include dapagliflozin, ertugliflozin, empagliflozin, canagliflozin, ipragliflozin, tofogliflozin, luseogliflozin, and remogliflozin [6]. The available evidence from preclinical studies and clinical studies showed that the SGLT2 inhibitors have distinctive morbidity and mortality reduction benefits in patients with type 2 diabetes mellitus (T2DM) and heart failure (HF). Accordingly, the European Association of the Study for Diabetes (EASD) and the American Diabetes Association (ADA), recommend SGLT2 inhibitors as the mainstay treatment of T2DM and the first line of T2DM treatment, in case of heart failure (HF) [7,8], and as an independent treatment of HF with a reduced ejection fraction, regardless of the diabetes status, according to the European Society of Cardiology (ESC) [9].

In addition to their blood glucose lowering effect, SGLT2 inhibitors have several pleiotropic benefits (Table 1), such as improving the visceral adiposity, reduction of body weight, lowering blood pressure, anti-inflammatory, anti-oxidant, as well as normalizing the serum uric acid levels and lipid profile [10,11,12,13]. Based on these reported benefits of the SGLT2 inhibitors, researchers are tempted to further examine the utility of these agents in the management of other diseases that are characterized by abnormal elevated levels of inflammation and oxidative stress, and more specifically, in neurological disorders.

## 2. Etiology and the Pathophysiology of Autism

The etiology of ASD is complex in nature. It could be associated with genetic factors and/or environmental components, such as infection, toxins, or medications, which in turn might induce several epigenetic changes [2]. Moreover, recent findings indicated a positive correlation between the brain development and the intestinal microbiota [29]. This explains why infants fed on cow’s milk formula had a drastically increased plasma osmolality which affected the homeostasis hemodynamics of the brain development negatively, compared to the breast-fed infants, emphasizing the gut microbiota-brain axis association to neurodevelopmental disorders [30,31]. The microbiome is essential for the microglial maturation process and taking control of the CNS glial activation, thus regulating the inflammation in the CNS, as the gut dysbiosis impacts the immune system homeostasis, which leads to developmental delays and to the developmental pathway disruption [32].

The risk of developing drug- induced ASD is increased during the second trimester of the fetal development, when exposed to neurotoxic or teratogenic drugs of various pharmacological classes of interest [33]. The pathological process of ASD remains unclear, but the neurological findings, during the first year of the child’s life, confirm the premature brain overgrowth, as a result of the excessive neuron numbers, which leads to defects in the neural wiring and patterning, with short cortical interactions hindering the function of the long-distance interactions within the brain regions in a large-scale. These networks underlie the communication and socio-emotional functions, such early alterations in the brain might be linked to the ASD clinical manifestations [34]. According to the literature, medications such as anticonvulsants and antidepressants have the potential of causing ASD during pregnancy, for instance, valproic acid (VPA) is an anticonvulsant drug used primarily in bipolar disorder and epilepsy [35]. During pregnancy, and at a vital stage of the nervous system development (second trimester), it was found to increase the risk of developing intellectual disorders, including ASD in children [35]. Moreover, preclinical studies showed that when rodents are prenatally exposed to VPA, they displayed neurodevelopmental characteristics which are comparable to those observed in the human setting [36]. When pregnant rodents were injected intraperitoneally (IP) to this drug on gestational day (GD) 12.5, the delivered pups were found to display social interaction impairments, anxiety, and recognition memory deficits, which are categorized as typical ASD-like behaviors [37,38,39]. Furthermore, it has been reported that VPA was capable of inducing dendritic spine loss in the prefrontal complex and the CA1 region of the hippocampus in the mouse model [40]. Furthermore, in a rat preclinical model, VPA was reported to significantly decrease the number of positive Nissl bodies in the lower layers of somatosensory cortex, as well as in the middle and lower layers of the prefrontal cortex (PFC), while it increased the apoptotic cell death and the histone levels in the neocortex [41,42]. Other medications, such as selective serotonin reuptake inhibitors (SSRIs) have been controversial in this case. An investigation which included 117,737 patients detected a significant association between SSRIs exposure across all trimesters and offspring ASD development [43,44], while it has been challenged by a retrospective study of 35,906 births confirming no relation between SSRIs exposure and ASD [45], which concludes that SSRIs exposure solely is not a confounding variable in causing ASD [46]. In addition to the behavioral testing, several biochemical assays showed that autistic children display elevated levels of plasma lipid peroxidation, reactive oxygen species (ROS), and a significant inhibition of antioxidant enzymes and ATP levels [47,48]. The mechanistic target of rapamycin (mTOR) is a substantial signaling node that receives input from the regulatory type proteins to send signals from the nutrient stores, the growth factors, energy, and the ambient oxygen levels [49]. Such involvements make mTOR a sensor for cell survival and growth cellular resources. Furthermore, mTOR has been implicated in the pyrimidine and purine nucleotide biosynthesis, the phosphorylation of other protein substrates, and the DNA transcription [50]. The mTOR enzyme plays a vital role in the brain, to establish the spine morphology, the axon development, the dendritic arborization, and the synaptic flexibility [51]. Brain malformations are associated with the mTOR regulatory genes mutations as the activation of the mTOR pathway in the hippocampal neurons, elevates the branching and growth of the dendritic arbors, while their complexity was decreased in such cells by the mTOR removal in vitro [52]. Signals of the mTOR complex 1 and the mTOR complex 2 were found to be crucial for a healthy dendritic arbor development of the rat hippocampus in vitro [53]. In mice models, mTOR have shown to play role in regulating the axon outgrowth in the mouse dorsal root ganglion, as it is upregulated after injury, which leads to the increased capacity of the axonal growth [54]. Moreover, the mTOR pathway regulates the balance in the brain between the cellular activation and the inhibition, thus the supply network integrity, the synaptic plasticity, and the learning capability [55]. Hence, numerous neurodevelopmental disorders are associated with the deviant mTOR pathway activation. Particularly, a subset of the cortical development malformations is directly caused by the mTOR activity regulators’ genes mutations [56]. An abnormal mTOR activity has been identified in brain development disorders, including the defective connectivity or synaptogenesis, such as epilepsy and ASD, and can be associated with intellectual disability [57]. Since abnormalities and dysregulations of mTOR have been identified in ASD; establishing this association would be of a significance from a therapeutic point of view, as the mTOR inhibitors are clinically available [58]. Accordingly, interventions that target these mechanistic pathways would plausibly impact the progression of the disease, thus improve the overall behavioral symptoms. Additionally, the preceding studies proposed a relation between the neurogenic disorders, including ASD and maternal infections [59]. Pregnant mice and rats infected with human influenza led to offspring with autistic behaviors [60]. Other viruses, such as the mumps, cyto-megalovirus, and measles are unlikely to be associated with current ASD cases because of the vaccination programs that effectively reduced their prevalence [61], while there is no evidence for vaccination to increase the risk of causing autism [62]. Toxic exposures, such as to heavy metals, pesticides, air pollutants, persistent, and non-persistent organic pesticides demonstrated a neurotoxicity by interacting with the genetics factors, hence modifying the neurodevelopment of the synapses, increasing the oxidative stress and neuroinflammation [63,64]. Genetic research discovered that the ASD etiology is robustly heterogenic and multigenic by sequencing technology, as hundreds of peril genes were identified, mainly those involved in the transcriptional regulation, chromatin remodeling, and synapse formation [65,66].

## 3. SGLT2 Inhibitors Decrease Oxidative Stress

**Figure 1:**
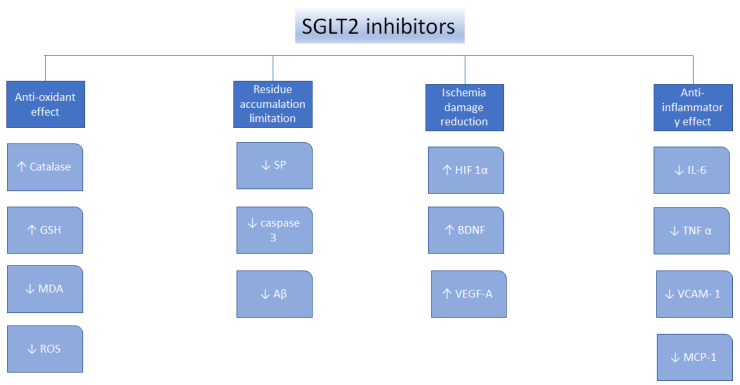
Mechanisms of action for the SGLT2 inhibitors. ↑—increase, ↓—decrease, glutathione—GSH, malondialdehyde—MDA, senile plaques—SP, amyloid β—Aβ, hypoxia-inducible factor 1α—HIF1α, brain-derived neurotrophic factor—BDNF, vascular endothelial growth factor A—VEGF-A, interleukin 6—IL-6, tumor necrosis factor α—TNFα, vascular cell adhesion protein—VCAM-1, reactive oxygen species—ROS, monocyte chemotactic protein-1—MCP-1 [75].

Oxidative stress, defined as the imbalance between the antioxidants production and the pro-oxidants levels, is a vital element underlying the pathogens of nephropathy, neural disorders, cardiovascular disorders, diabetes mellitus, liver conditions, and cancer [67]. SGLT2 inhibitors were found to perform as antioxidants indirectly because of their ability to reduce the generation of free radicals [68], to upregulate the antioxidant systems, such as glutathione (GSH) and superoxide dismutase (SOD) [69,70,71], to suppress pro-oxidants, such as thiobarbituric acid-reactive substances (TBARS), to reduce nicotinamide adenine dinucleotide phosphate oxidase 4 (NOX4) [72,73], and decrease the glucose-induced oxidative stress [74]. Moreover, canagliflozin, dapagliflozin, and empagliflozin were found to decrease oxidative stress in many types of cancer, by suppressing the cellular proliferation [75,76,77,78,79] (Figure 1).

## 4. The Anti-Inflammatory Characteristics of the SGLT2 Inhibitors

**Figure 2:**
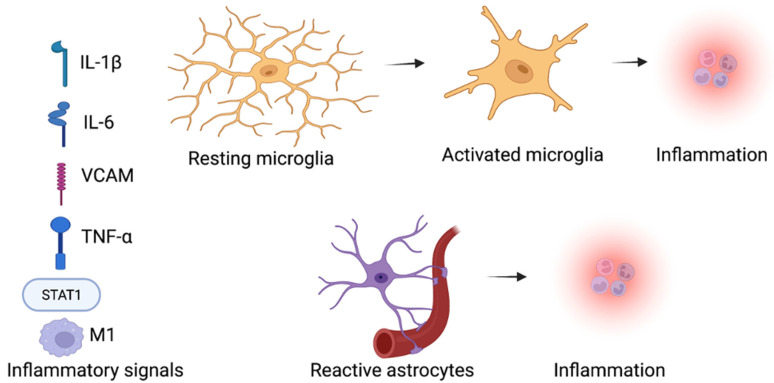
Inflammatory signals promote inflammation by activating the microglia and astrocytes within the brain in ASD. SGLT2 inhibitors influence on the inflammation and neuroinflammation, SGLT2 inhibitors decrease the inflammatory factors levels, such as the M1 macrophages, STAT1 inflammatory transcription factor, cytokine interleukin-1β (IL-1β), tumor necrosis factor (TNF-α), and vascular cell adhesion protein (VCAM) in neurodevelopmental diseases [43,44]. (Created with BioRender.com).

The process of inflammation has a significant role in neurodevelopmental diseases, metabolic diseases, lifelong kidney disorder, liver disease, cardiovascular disease, and cancer [80,81]. SGLT2 inhibitors have shown anti-inflammatory effects in multiple preclinical disease models [82]. SGLT2 inhibitors have been reported to downregulate the pro-inflammatory mediators, including transforming the growth factor beta (TGF-β), interleukin-6 (IL-6), c-reactive protein (CRP), nuclear factor κB (NF-κB), tumor necrosis factor alpha (TNF-α), and the monocyte chemoattractant protein (MCP-1) [83,84]. The SGLT2 inhibitors were found to attenuate the inflammation by their capability to regulate the imbalanced redox state, the tissue hemodynamic alterations, and the renin-angiotensin system (RAS) [85,86]. Furthermore, the expression of proinflammatory cytokines could be the result of the activated transcription factors activated by oxidative stress, including the nuclear factor erythroid 2–related factor 2 (Nrf2), peroxisome proliferator-activated receptor (PPAR) γ, hypoxia-inducible factor (HIF)-1α, and NF-κB [87,88]. SGLT2 inhibitors reduced oxidative stress in many diseases, and they may also reduce inflammation caused by the chemokines regulation and cytokines transcription factors (Figure 2).

## 5. Pleiotropic Perspective of the SGLT2 Inhibitors in ASD

The effects of the SGLT2 inhibitors have been extensively studied [6,10,13,89,90,91], and the assessment of the design, limitations, and outcomes of the SGLT2 inhibitors trials have been comprehensively discussed [6,92,93]. Different from other reviews on SGLT2 inhibitors, this review aims thoroughly at focusing on the studies that assessed the SGLT2 inhibitors benefits from a neurodevelopmental point-of-view. Databases, such as PubMed, MEDLINE, and Google Scholar have been searched using the following terms: SGLT2 inhibitors, antioxidants, oxidative stress, and neurodevelopmental diseases, up to 31 January 2022. This review will discuss how the SGLT2 inhibitors regulate the ASD biomarkers, based on the clinical evidence supporting their function as antioxidants and anti-inflammatories that can cross the blood-brain barrier (BBB). This disease-specific review will provide a better understanding of the potential of antioxidant and anti-inflammatory roles of the SGLT2 inhibitors in ASD. The potential therapeutic role of the SGLT2 inhibitors in ASD have not yet been studied. Therefore, this review aims at providing the theoretical evidence about their plausible efficacy in these disorders, which would encourage further research in this area.

## 6. Role of the SGLT2 Inhibitors in Neurodevelopmental Disorders

Glucose is the primary metabolic substrate of the neural function [94]. It is transported across the BBB into the brain and made available to the glial cells and neurons via several glucose transporters (GLUTs) [95]. Of notable importance in the CNS, are the GLUT1 which is expressed in the glial cells and BBB, and the GLUT3 which is expressed in the neurons [96]. SGLT receptors were originally found to be expressed in the intestines and kidneys, but later protein expression studies showed that the SGLT receptors are distributed in different brain areas, such as the hippocampus, putamen, frontal cortex, hypothalamus, parietal cortex, and brainstem [97]. Moreover, it has been shown that the SGLT2 inhibitors can cross the BBB as they are partially lipid soluble. Accordingly, numerous studies have underlined the protein expression of the SGLT2 receptors in the encephalon of APP/PS1xd/db mice, emphasizing their potential therapeutic benefits in neurodevelopmental disorders [98,99].

### 6.1. SGLT2 Inhibitors in Diabetes Mellitus and Ischemic Stroke

Diabetes mellitus and obesity are risk factors for cognitive disorders [100]. It has been reported that a dose of 10 mg/kg of empagliflozin given for a period of 22 weeks, notably improved the cognitive status of db/db mice, by increasing the cerebral brain derived neurotrophic factor and the reduction of the cerebral oxidative stress [101]. In addition, a previous study has shown that receiving of 10 mg/kg of empagliflozin 24 h following a reperfusion, has decreased the parenchymal microglial burden of APP/PS1xdb/db mice and db/db mice [102]. Another preclinical study showed that the exposure to 10 mg/kg of empagliflozin at 24-hour following the reperfusion, ameliorated the neurological obstruction in ischemic stroke rat model in a dose-dependent manner [103]. Similarly, a decrease in the malondialdehyde (MDA) levels, elevated the catalase activity, and increased the GSH levels were observed in rat brain cells, following the systemic administration of empagliflozin leading to the suppressed levels of inflammation and the cerebral oxidative stress [103].

### 6.2. SGLT2 Inhibitors’ Ameliorative Impact in AD and PD

Parkinson’s disease (PD) and Alzheimer’s disease (AD) are common age-related neurodegenerative conditions. Currently, the available pharmacotherapeutic options for AD and PD merely provide symptomatic relief without curing the process [104]. The main pathological characteristics of AD, are the intra- and extra-cellular plaques accumulation, which is composed of neurofibrillary tangles (NFTs) and beta-amyloid. Recent studies have stated that the inhibition of the SGLT receptors may provide a beneficial effect on this process. A previous APP/PS1xdb/db mouse model study reported that empagliflozin reduced the insoluble and soluble levels of amyloid β in the hippocampus and the cortex of tested mice, with an overall reduction of the senile plaque density [102]. Moreover, in the scopolamine-induced memory impairment in the rat model, canagliflozin was found to prevent the memory impairment [105]. Such observed therapeutic effects might be attributed to the inhibition of the acetylcholinesterase enzyme, which is a property of canagliflozin and other SGLT2 inhibitors [66]. Furthermore, several previous studies have shown that the oxidative stress-induced neuroinflammation can lead to a mitochondrial dysfunction and glial cell activation, causing multiple molecular events in the brain, including the neural cell dissolution in PD and AD [106]. In a recent preclinical study involving a rotenone-induced PD in a rat, it was shown that a dose of 150 mg/kg of dapagliflozin was able to enhance PD motor activity in the rotarod and open-field tests [106]. In addition, dapagliflozin at the same dose, was found to alleviate the neuronal oxidative stress, through the reduction of the lipid peroxides [106]. Furthermore, dapagliflozin was reported to upregulate the glial derived neurotrophic element and its phosphatidylinositol 3-kinase protein kinase, and the glycogen synthase kinase-3β (GSK-3β) pathway, which affects the regulation of numerous key cellular operations, such as proliferation, senescence, differentiation, motility, and survival [106].

### 6.3. Application of the SGLT2 Inhibitors in Epilepsy

Erdogan et al. 2018 illustrated that the activity of a pentylenetetrazol-induced seizure in a rat model was decreased significantly, following exposure to the SGLT2 inhibitor dapagliflozin in a dose-dependent manner (75–150 mg/kg, i.p.), which has been attributed to the reduction in the sodium transport across the neuronal membranes, and the decreased glucose availability, hence stabilizing against the depolarization and excitability [107]. Collectively, the impact of the SGLT2 inhibitors in the neural disorders have shown promising therapeutic potentials, and some of the most significant observations are summarized in Table 2.

## 7. SGLT Receptors as a Potential Target for Neurological Disorders

**Figure 3:**
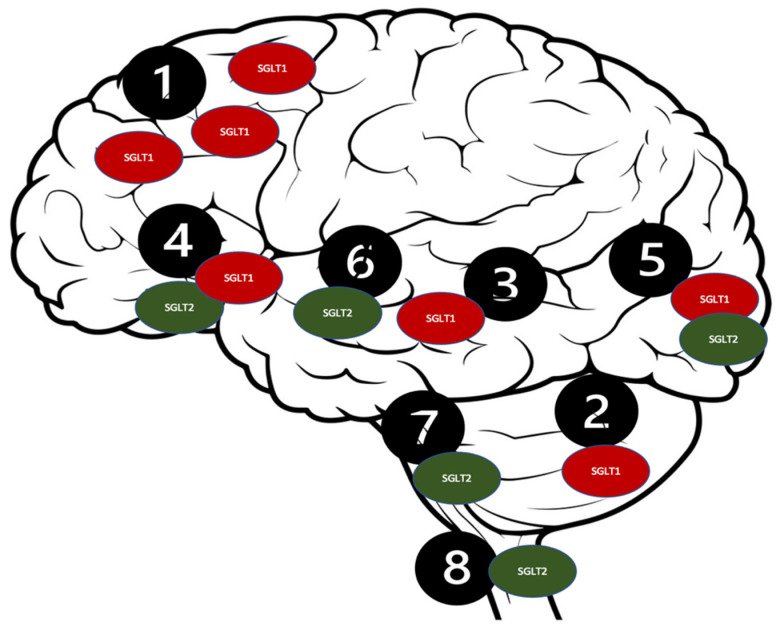
Distribution of the SGLT receptors in the CNS. 1. Brain cortex (pyramidal cells); 2. Purkinje neurons; 3. Hippocampus; 4. Hypothalamus; 5. Micro vessels; 6. Amygdala cells; 7. Periaqueductal gray; 8. Dorsomedial medulla.

SGLT co-transporters contain spanning monomer proteins, including a single *N*-glycosylation site and 14 transmembrane domains. They transport galactose and glucose against a concentration gradient alongside the simultaneous Na^+^ ions transportation [108]. Numerous reports have stated the presence of the SGLT receptors in a mammalian central nervous system (CNS) [97,109]. Furthermore, studies have proven that the SGLT2 receptors are significantly expressed in the cerebellum, the BBB endothelial cells, and the hippocampus, while the SGLT1 receptors are expressed in the CA1 and CA3 regions of the hippocampus [110,111,112,113] (Figure 3).

Such a distribution of the SGLT2 receptors [114] could potentially be responsible for their intriguing neuroprotective qualities, which could be beneficial in several neurological disorders, including ASD [99]. The SGLT2 inhibitors’ proposed mechanisms are presented in Figure 3. The antioxidant effect of the SGLT2 inhibitors can be attributed to their stimulatory action on the nuclear factor erythroid 2 (Nrf2)- related factor 2 pathway [115]. This displays the antioxidant activity because of their genetic expression of the antioxidant proteins, including glutathione-s-transferase (GST), SOD, and NADPH quinone dehydrogenase-1 to protect against cellular apoptosis [116]. The anti-inflammatory characteristics of the SGLT2 inhibitors could be accredited to the downregulation of NF-KB, which decreases IL-1β and the TNF-α expression [117]. Empagliflozin has the highest selectivity for the SGLT2 receptors (2500-fold) when compared to dapagliflozin which has (1200-fold) selectivity, and canagliflozin (250-fold) [118,119]. Therefore, in the context of the neuroprotective effects associated with the SGLT1 and SGLT2 receptors’ inhibition, canagliflozin was hypothetically preferred over other SGLT2 inhibitors, due to its dual SGLT1/SGLT2 inhibition capability [120].

## 8. Mutual Oxidative Biomarkers of ASD and the Potential Therapeutic Utility of the SGLT2 Inhibitors

ASD is a neurodevelopmental disorder characterized by diverse range of the impaired social abilities and communication, stereotypic and repetitive behaviors [121]. ASD could be diagnosed in early childhood, with a female-to-male ratio of 1:4, and an increasing prevalence over the past 20 years, as the current estimate is one in 160 children worldwide has an ASD [2,122]. There is no definitive curative medication for ASD yet, as the currently available pharmacological or behavioral therapies cannot enhance the core parameters of ASD [123,124,125]. Early detection of the autism symptoms is crucially impacting children’s adaptive skills development, and social intelligence, which have been reported in many studies practicing advanced imaging methods [126]. Finding validated biomarkers for the ASD screening or treatment follow-up have been attempted in many prospective studies, as the Autism Birth Cohort, has considered the proteomic, genetic, metagenomic, microbiological, and immunological parameters to utilize in a case-control study [127]. Moreover, the 1-Year Well-Baby Check-up Approach investigated various endpoints in autistic patients, including the gene expression, the immune system functionality, early brain growth, and cerebellar functions, to identify the biomarkers of the disease [128]. In neurodevelopmental diseases, such as ASD, oxidative stress was found to change the intracellular balance between the antioxidant defense mechanisms and the reactive oxygen species (ROS) [129]. Accordingly, both the enzymatic and nonenzymatic mechanisms of protection have been reported to exist. Enzymes include catalase (CAT), SOD, ceruloplasmin, and glutathione peroxidase (GPX) [130]. In contrast, other systems include phenolic compounds, glutathione (GSH), and the antioxidant nutrients (vitamins, A C, E, B6; folate) [131]. Moreover, elevated oxidative stress was found to trigger the activation of the mast cell, which in turn increases the production of the proinflammatory, neuro-sensitizing, and vasoactive molecules connected to ASD, such as histamine, IL-6, bradykinin, tryptase, and the vascular endothelial growth factor (VEGF) [131]. These factors interrupt the BBB [132], permitting the entero-toxic molecules into the brain inducing neuroinflammation [133]. Oxidative stress has a central function in the ASD pathogenesis as it upregulates the neural deterioration in the genetically susceptible patients. The mammalian brain reacts to oxidative stress deterioration, as it accounts for 20% of the basal oxygen consumption, whereas it is responsible for a few percent of body weight [134]. Numerous animal studies have assessed the impact of the oxidative stress on the CNS in different models [135,136,137]. The imbalance between the antioxidant and oxidant systems involved in the neurodegenerative pathologies, e.g., AD [138], and mental disorders, including bipolar disorder and schizophrenia [139,140,141]. In a postmortem analysis study, brain tissues from autistic patients were found to display higher levels of oxidative stress biomarkers than healthy individuals [142,143,144,145,146]. The association between ASD and oxidative stress was disclosed in various studies with different biomarkers, as illustrated in Table 3, head-to-head with the mutual antioxidative ability of the SGLT2 inhibitors [147,148].

SGLT2 inhibitors have the potential to improve ASD patients’ behavioral and brain disruptions by increasing the cerebral brain derived neurotrophic factor and reducing the cerebral oxidative stress, including elevated the GSH and catalase activity, reduced MDA, amyloid β levels, plaque density, and acetylcholinesterase [101,102,103,104].

## 9. Conclusions

ASD remains a global health dilemma, as it is a chronic condition, and is incurable, leading to a reduced quality of life. It is crucial to find the mutual molecular mechanisms of ASD and redefine the indications for the well-studied medication with numerous pleiotropic effects to find a solution. This review has disclosed the impact of the SGLT2 inhibitors in neurological diseases, which could relate to ASD as it shares multiple pathways and mutual biomarkers. SGLT2 inhibitors display several neuroprotective properties, highlighting their therapeutic potential for ASD patients, as these agents have the capability to inhibit the acetylcholinesterase enzyme, reduce the elevated levels of the oxidative stress in the brain, and restore the anabolism and catabolism balance. Moreover, clinical intervention studies are vital to determine whether the displayed methods are useful as the SGLT2 inhibitors have never been tested on ASD directly. Currently, our research team is conducting a preclinical experiment to assess the effects of canagliflozin on the VPA-induced ASD in Wistar rats.

## Figures and Tables

**Table 1:** Comparison between the SGLT2 inhibitors affinity and pleotropic effects.

	Sotagliflozin	Canagliflozin	Dapagliflozin	Empagliflozin	Ertugliflozin
Affinity for SGLT2 over SGLT1	20 fold [14]	250 fold [14] (Dual inhibitor)	1200 fold [14]	2500 fold [14]	2500 fold [14]
AChE inhibition	K_i_ 5.6 µM [15]	K_i_ 0.13 µM (most potent) [15]	K_i_ 25.02 µM [15]	K_i_ 0.177 µM [15]	K_i_ 31.69 µM [15]
Anti-inflammatory	Not applicable	Yes [16]	Yes [17]	Yes [18]	No [19]
Oxidative stress inhibition	Yes [20]	Yes [21]	Yes [22]	Yes [23]	Yes [24]
Nervous system remodeling	Not applicable	Not applicable	Not applicable	Yes [25]	Not applicable
mTOR signaling reduction	Not applicable	Yes [26]	Yes [26]	Yes [27]	Yes [28]

**Table 2:** Preclinical evidence of the reparative implications of the SGLT2 inhibitors in neurological disorders.

Disorder	Animal Species	Medication	Results	References
Cognitive impairment	Mice	Empagliflozin	Increase the cerebral brain derived neurotrophic factor and reduce the cerebral oxidative stress.	[61,62]
AD	Rats	Canagliflozin	Reduce amyloid β levels, plaque density, and acetylcholinesterase.	[66]
PD	Rats	Dapagliflozin	Upregulate the GDNF/PI3K/AKT/GSK-3β pathway and reduce the ROS-dependent neuronal apoptosis.	[67]
Epilepsy	Rats	Dapagliflozin	Reduce sodium and glucose transported across the neurons.	[68]
Stroke	Rats	Empagliflozin	Upregulate VEGF and HIF-1α; decreased MDA, elevated GSH and activity of catalase.	[63,64]

**Table 3:** Comparison of the oxidative stress biomarkers’ blood levels in autistic patients with a mutual therapeutic biomarker impact of the SGLT2 inhibitors. ↑—increase, ↓—decrease.

ASD Biomarkers	Result	Reference	SGLT2 Inhibitor Name/Subject of Study	Result	Reference Number
GSH	Statistically significantly lower level of GSH in the ASD group than in the control group.	[148,149,150,151]	Empagliflozin/Wistar rats	↑ GSH	[103]
CAT	Lower CAT activity in the erythrocytes of autistic patients than in the healthy controls.	[152,153]	Empagliflozin/Wistar rats	↑ CAT	[103]
GPX	GPX activity in the erythrocytes is significantly lower in the ASD group than in the control group after the meta-analysis.	[152,154,155,156,157]	Dapagliflozin/Wistar rats	↑ GPX	[158]
TNF-α	ASD children produced more TNF-α than those obtained from the control.	[159,160,161]	Empagliflozin/ApoE-/-mice	↓ TNF-α	[83]
IL-6	Autistic mice displayed elevated IL-6 in the brain.	[155,156,157,158,159,160,161,162]	Empagliflozin/ApoE-/-mice	↓ IL-6	[83]
Caspase-3	Assessed the active caspase-3 levels and determined the significant elevation in children with ASD.	[156,157,158,159,160,161,162,163,164,165,166]	Empagliflozin/Wistar rats	↓ caspase 3	[102]
HIF-1α	Serum HIF-1α levels were borderline significantly lower in the ASD group.	[167]	Empagliflozin/Wistar rats	↑ HIF-1α,	[102]
Aβ	Severe ASD patients produced beta-amyloid at twice more than the control group and four times more than the mild ASD group.	[168,169]	Empagliflozin/APP/PS1xdb/db mice	↓ Aβ	[101]

## Data Availability

The data in this study were not created nor analyzed by the authors. Data sharing is not applicable to this article.

## Abbreviations

ADA	American Diabetes Association
AD	Alzheimer’s disease
APP/PS1	Amyloid precursor protein double transgenic mice expressing a chimeric mouse/human (Mo/HuAPP695swe) and a mutant human presenilin 1
ASD	Autism spectrum disorder
BBB	Blood-brain barrier
CNS	Central nervous system
CRP	C-reactive protein
db/db	Diabetes mellitus most widely used mouse model
EASD	European Association of the Study for Diabetes
ESC	European Society of Cardiology
FDA	Food and Drug Administration
GD	Gestational day
GLUTs	Glucose transporters
GSH	Glutathione
GST	Glutathione-s-transferase
GSK-3β	Glycogen synthase kinase-3β
HF	Heart failure
HIF-1α	Hypoxia-inducible factor
IL-6	Interleukin-6
i.p	Intraperitoneally
MDA	Malondialdehyde
mTOR	Mechanistic target of rapamycin
MCP-1	Monocyte chemoattractant protein
NFTs	Neurofibrillary tangles
NADPH	Nicotinamide adenine dinucleotide phosphate-reduced
NOX4	Nicotinamide adenine dinucleotide phosphate oxidase 4
Nrf2	Nuclear factor erythroid 2–related factor 2
NF-κB	Nuclear factor κB
PD	Parkinson’s disease
PFC	Prefrontal cortex
PPAR	Peroxisome proliferator-activated receptor
ROS	Reactive oxygen species
RAS	Renin-angiotensin system
SSRIs	Selective serotonin uptake inhibitors
SGLT2	Sodium-glucose cotransporter 2
SOD	Superoxide dismutase
TBARS	Thiobarbituric acid-reactive substances
TNF-α	Tumor necrosis factor alpha
T2DM	Type 2 diabetes mellitus
VPA	Valproic acid
VEGF	Vascular endothelial growth factor
